# Association of anti-Ro-52 antibodies with occurrence of interstitial lung disease in patients with idiopathic inflammatory myopathy

**DOI:** 10.1186/s13075-024-03382-x

**Published:** 2024-08-22

**Authors:** Chia-Tse Weng, Tang-Hsiu Huang, Chun-Hsin Wu, Yuan-Ting Sun

**Affiliations:** 1 Division of Allergy, Immunology and Rheumatology, Department of Internal Medicine, College of Medicine, National Cheng Kung University Hospital, National Cheng Kung University, No. 138, Sheng-Li Road, Tainan, 704 Taiwan; 2 Division of Chest Medicine, Department of Internal Medicine, College of Medicine, National Cheng Kung University Hospital, National Cheng Kung University, No. 138, Sheng-Li Road, Tainan, 704 Taiwan; 3 Department of Neurology, College of Medicine, National Cheng Kung University Hospital, National Cheng Kung University, No. 1, University Road, Tainan, 701 Taiwan; 4 Department of Medical Genomics, College of Medicine, National Cheng Kung University Hospital, National Cheng Kung University, No. 138, Sheng-Li Road, Tainan, 704 Taiwan

**Keywords:** Idiopathic inflammatory myopathy, Interstitial lung disease, Anti-Ro-52 antibody, Anti-aminoacyl-tRNA synthetase antibody

## Abstract

**Background:**

Anti-Ro-52 antibodies have been associated with interstitial lung disease (ILD) in various autoimmune diseases. However, their role in ILD among patients with idiopathic inflammatory myopathies (IIMs) is relatively underexplored. This study aimed to investigate the association between anti-Ro-52 antibodies and the occurrence of ILD in individuals with IIMs.

**Methods:**

This retrospective observational study included 604 patients who underwent myositis autoantibody testing between July 2018 and January 2021 at our hospital and were diagnosed with either IIMs or IIM-mimics. Comparative analyses were conducted between IIMs and IIM-mimics, as well as within the IIM group between cases with and without ILD. Logistic regression or Firth’s logistic regression analyses were employed to assess the risk of ILD development in different IIM subgroups and myositis antibody categories.

**Results:**

This study included 190 patients with IIM and 414 patients with IIM-mimics. Patients with IIM demonstrated higher incidence of ILD, concurrent autoimmune disease, and a greater likelihood of various myositis autoantibodies when compared to the IIM-mimics group. Within the IIM patient cohort, those with ILD exhibited a later age of onset of IIM, an increased mortality rate, and a more frequent presence of anti-aminoacyl-tRNA synthetase (ARS) antibodies compared to those without ILD. The presence of any myositis-specific antibody (MSA) was associated with a six-fold increased risk of ILD, while dual positivity for MSA and anti-Ro-52 antibodies conferred a twenty-fold risk. Anti-ARS antibodies carried a 14-fold increased risk of ILD, which escalated to 38-fold in cases of dual positivity for anti-ARS and anti-Ro-52 antibodies. Anti-Ro-52 antibodies alone increased the risk eight-fold.

**Conclusions:**

Among patients with IIM, the presence of ILD was linked to higher mortality. Certain autoantibodies, notably anti-ARS and anti-Ro-52 antibodies, were associated with an increased risk of ILD. The greatest risk of ILD was observed in cases of dual positivity for anti-ARS and anti-Ro-52 antibodies.

**Supplementary Information:**

The online version contains supplementary material available at 10.1186/s13075-024-03382-x.

## Introduction

Idiopathic inflammatory myopathies (IIMs), commonly referred to as myositis, constitute a diverse array of autoimmune disorders characterized by heterogeneity in clinical manifestations, treatment responses, and prognoses. While muscle weakness typically serves as the hallmark of these conditions, they can also impact various other organs such as the skin, joints, lungs, heart, and gastrointestinal tract, which vary in severity and frequency among different subtypes of IIMs. In some instances, the disease may predominantly manifest in these extramuscular domains. This systemic involvement highlights the comprehensive inflammatory nature of IIMs [1, 2].

Traditionally, IIMs have been classified into three main subtypes: polymyositis (PM), dermatomyositis (DM), and inclusion body myositis (IBM). However, in recent years, other forms of IIMs, i.e., immune-mediated necrotizing myopathy (IMNM) and antisynthetase syndrome (ASS), have been recognized as distinct subtypes based on their unique clinical, pathological, and immunological features [3, 4]. ASS is characterized by the presence of antisynthetase antibodies, which target aminoacyl-tRNA synthetases, and is associated with a unique set of clinical features such as interstitial lung disease (ILD), fever, arthritis, mechanic’s hand, and Raynaud’s phenomenon [5]. While ASS shares many features with PM and DM, its unique clinical and immunological characteristics justify its classification as a separate subtype of IIMs [1, 4].

Myositis-specific antibodies (MSAs) are a group of autoantibodies that are highly specific to certain subtypes of IIM [6]. They have helped to refine the classification and diagnosis of these disorders [1, 2, 6]. For example, the identification of antisynthetase antibodies has led to the recognition of ASS as a distinct subtype of IIMs [4, 5]. Similarly, the discovery of other MSAs, such as anti-melanoma differentiation-associated protein-5 (anti-MDA5) and anti-TIF1γ, has led to the identification of new subtypes of dermatomyositis with distinct clinical and prognostic features [7, 8]. Thus, the identification of MSAs has allowed for more accurate diagnosis and classification of IIMs, leading to better management and treatment options for affected individuals.

Given its high prevalence and mortality rate, interstitial lung disease (ILD) is considered the most significant extramuscular manifestation of IIM [9, 10]. There are several risk factors associated with the development of ILD in patients with IIMs, such as older age at diagnosis, arthritis/arthralgia, fever, elevated erythrocyte sedimentation rate and C-reactive protein level, presence of anti-Jo-1 antibodies, and presence of anti-MDA5 antibodies [11, 12]. Certain subtypes of IIMs, such as anti-MDA5 dermatomyositis and ASS, have a higher risk of developing ILD compared to other subtypes [6, 13]. Therefore, identifying these risk factors is important for managing and treating ILD in patients with IIM.

Myositis-associated antibodies (MAAs) are a group of autoantibodies frequently identified in patients with IIMs and have also been detected in patients with other connective tissue diseases (CTDs), in which myositis can occur [1, 6]. The prominent MAAs in this category comprise anti-Ro-52, anti-PM-Scl, anti-Ku, and anti-U1RNP antibodies [1, 6]. Ro-52, also known as TRIM21, is a multifunctional protein that belongs to the tripartite-motif (TRIM) family, playing a pivotal role in various cellular processes, including immunity and protein degradation. After extensive research, Ro-52 has been differentiated from Ro-60 in the Ro/SSA system family [14]. Anti-Ro-52 antibodies are frequently identified in the serum of patients suffering from a range of autoimmune disorders, including but not limited to primary Sjögren’s syndrome, systemic lupus erythematosus (SLE), systemic sclerosis (SSc), mixed connective tissue disease (MCTD), myositis, autoimmune hepatitis, and primary biliary cirrhosis [15]. They have also been implicated as a risk factor for ILD in patients with these various autoimmune diseases, including SSc, Sjögren syndrome, and MCTD [16–19]. However, there is limited research on the role of anti-Ro-52 antibody as a risk factor for ILD in patients with IIM [20–22]. Therefore, the aim of this study was to investigate the association between anti-Ro-52 antibodies and the occurrence of ILD in patients with IIM.

## Method

### Study cohort and data collection

This retrospective observational study was conducted at the National Cheng Kung University Hospital, involving 604 patients who underwent myositis autoantibody testing between July 2018 and January 2021. The patients were diagnosed with either IIMs or IIM-mimics, based on a combination of clinical presentations, muscle enzymes, electromyography, autoantibody status, muscle magnetic resonance imaging, and muscle or skin biopsy. We retrieved clinical and laboratory data from the medical records, including demographic information such as age of onset and gender, as well as laboratory results such as creatine kinase (CK), lactate dehydrogenase (LD), and aspartate aminotransferase (AST) levels, and autoantibody status. Additionally, we recorded the presence of concurrent autoimmune diseases, malignancy, mortality, and various clinical features such as ILD, muscle weakness, skin rash, and more. The study protocol received approval from the Institutional Review Board (IRB) of the National Cheng Kung University Hospital (Serial No.: A-ER-108-071, B-ER-109-120). Due to the retrospective design of the study, the IRB waived the need for informed patient consent.

### Classification of IIM and definition of IIM-mimics

The patients with IIM were further classified into six subgroups: DM, PM, ASS, IMNM, IBM, and overlap myositis. This classification was determined based on a combination of factors including the presence of myositis autoantibodies, pathological findings from muscle or skin biopsies, and clinical symptomatology. The diagnoses of DM and PM were made in accordance with the Bohan and Peter criteria [23, 24], whereas many patients previously identified as having PM are categorized within emerging subgroups like ASS or IMNM. Anti-synthetase syndrome (ASS) was defined by the presence of anti- aminoacyl-tRNA synthetase (ARS) autoantibodies along with one or more of the following criteria: evidence of myositis according to the Bohan and Peter criteria, evidence of interstitial lung disease (ILD), signs of arthritis, unexplained fever, Raynaud phenomenon, or the presence of mechanic’s hands [25]. Accordingly, myositis was not mandatory for the diagnosis of ASS. IMNM was diagnosed based on the 2016 European Neuromuscular Centre (ENMC) criteria, which may not necessitate a muscle biopsy [3, 26]. IBM diagnosis was made using the ENMC IBM Research Diagnostic Criteria 2011 [27]. Overlap myositis was defined as myositis that coexists with another CTD, such as primary Sjögren’s syndrome, SLE, or SSc. The diagnosis of ILD was established based on findings from chest X-rays and/or chest computed tomography scans. IIM-mimics are defined as medical conditions or illness that exhibit certain clinical features resembling those of IIMs but are not authentic IIMs themselves. These conditions often manifest symptoms such as muscle weakness, myalgia, skin rashes, ILD, or other characteristics commonly observed in IIMs. This similarity in clinical presentation can initially create diagnostic uncertainty and prompt healthcare providers to order myositis antibody studies to differentiate between IIMs and these mimic conditions.

### Detection of myositis autoantibodies

Our hospital employs a commercial immunoblot assay, the “Autoimmune Inflammatory Myopathies 16 Ag” panel (Euroimmun, Lubeck, Germany), to detect a total of sixteen MSAs and MAAs. The MSAs being detected encompass antibodies against Mi-2α, Mi-2β, TIF-1γ, MDA5, NXP2, SAE, SRP, Jo-1, PL-7, PL-12, EJ, and OJ, while the MAAs being detected include antibodies targeting Ku, PM-Scl 100, PM-Scl 75, and Ro-52. The positivity of these antibodies was determined using a semi-quantitative approach, where grades such as moderate positive (++) and strong positive (+++) indicated positive results in this study. Results categorized as weak positive (+), borderline (±), or negative (−) were considered negative outcomes.

### Statistical analysis

The collected data were analyzed using descriptive statistics, such as median, interquartile range (IQR), mean, and standard deviation. Statistical analyses were performed to compare the two groups. Independent samples t-test or Mann-Whitney U-test were used for continuous variables, depending on the data distribution and assumptions. Chi-squared test and Fisher’s exact test were used for categorical variables, based on sample size and test assumptions. Furthermore, logistic regression or Firth’s bias-reduced penalized-likelihood logistic regression were employed to calculate odds ratios (ORs) and 95% confidence intervals (CIs) for assessing the risk of ILD development in various IIM subgroups and myositis antibody categories. Statistical significance was set at a *p*-value of < 0.05. The statistical analysis was conducted using SAS version 9.4 (SAS Institute, Cary, NC, USA).

### Sensitivity analysis for autoantibodies

Sensitivity analyses were performed to evaluate the diagnostic efficacy of autoantibodies, specifically MSAs and anti-ARS antibodies, in discriminating between cases of IIM and IIM-mimicking conditions. Two distinct models, denoted as Model 1 and Model 2, were employed for this assessment. In Model 1, autoantibodies were considered positive if they exhibited titers of moderate positive (++) and strong positive (+++), as described previously. Conversely, in Model 2, autoantibodies were considered positive if they displayed titers of weak positive (+), moderate positive (++), or strong positive (+++). These sensitivity analyses aimed to provide valuable insights into the potential of autoantibodies for aiding in the differentiation of IIM cases from IIM-mimics under varying criteria for positivity.

## Results

### Clinical characteristics of study participants

The study included 190 patients with IIM and 414 patients with IIM-mimics (Supplementary Table 1). The age of onset and the percentage of male patients were similar in both groups. The presence of concurrent autoimmune diseases was more common in the IIM group than in the IIM-mimic group. The IIM group also had a significantly higher incidence of ILD compared to the IIM-mimic group (46.84% vs. 21.26%, *p* < 0.001). There were no significant differences in malignancy or mortality rates between the two groups.

Although the median CK levels were higher in the IIM group, the difference was not statistically significant (*p* = 0.363). However, the median levels of LD and AST were significantly higher in the IIM group than in the IIM-mimic group (both *p* < 0.001). Regarding the prevalence of MSAs and MAAs, there were significant differences between the two groups. Patients with IIM were more likely to have TIF1γ, MDA5, SRP, Jo-1, PL-7, PL-12, and EJ antibodies compared to IIM-mimics. Anti-Ro-52 antibodies were also significantly more common in the IIM group compared to the IIM-mimics group (*p* < 0.001 and *p* = 0.002, respectively).

### Clinical characteristics of IIM patients with and without ILD

There was a total of 190 patients with IIM in the study, with 89 (46.84%) of them having ILD and 101 (53.16%) without ILD (Table 1). The mean age of onset was significantly higher in patients with ILD (56.6 years) than those without ILD (49.0 years) with a *p*-value of < 0.001. There was no significant difference in gender between the two groups. The median CK levels were higher in patients without ILD (585.5 U/L) than those with ILD (389.0 U/L), but this difference was not statistically significant. The median LD and AST levels were similar between the two groups.

**Table 1:** Clinical and serological features of 190 patients with IIM stratified by ILD

	ILD(+)	ILD(-)	*p*-value
n (%)	89 (46.84)	101 (53.16)	
Age of onset (years), mean (SD)	56.6 (12.7)	49.0 (16.7)	< 0.001
Male, n (%)	25 (28.09)	34 (33.66)	0.502
CK (U/L), median (IQR)	389.0 (113.0, 1687.5)	585.5 (151.0, 4021.0)	0.100
LD (U/L), median (IQR)	367.0 (264.0, 538.0)	360.0 (244.0, 662.0)	0.997
AST (U/L), median (IQR)	60.0 (35.0, 159.0)	69.0 (38.0, 204.0)	0.472
IIM subgroup, n (%)			< 0.001
DM	19 (21.35)	33 (32.67)	
ASS	56 (62.92)	7 (6.93)	
IMNM	1 (1.12)	15 (14.85)	
PM	1 (1.12)	13 (12.87)	
Overlap myositis	12 (13.48)	32 (31.68)	
IBM	0	1 (0.99)	
Concurrent autoimmune diseases, n (%)			0.494
None	59 (66.29)	64 (63.37)	
Systemic lupus erythematosus	3 (3.37)	12 (11.88)	
Primary Sjögren’s syndrome	13 (14.61)	10 (9.90)	
Systemic sclerosis	3 (3.37)	4 (3.96)	
Rheumatoid arthritis	5 (5.62)	5 (4.95)	
Mixed CTD	1 (1.12)	1 (0.99)	
Undifferentiated CTD	5 (5.62)	5 (4.95)	
Malignancy, n (%)	8 (8.99)	10 (9.90)	1.000
Mortality, n (%)	12 (13.48)	3 (2.97)	0.016
MSAs, n (%)			
Mi-2α	0 (0.00)	4 (3.96)	0.124
Mi-2β	1 (1.12)	3 (2.97)	0.624
TIF1γ	1 (1.12)	11 (10.89)	0.014
MDA5	8 (8.99)	4 (3.96)	0.261
SAE1	1 (1.12)	1 (0.99)	1.000
NXP2	4 (4.49)	1 (0.99)	0.188
SRP	1 (1.12)	10 (9.90)	0.023
Jo-1	23 (25.84)	5 (4.95)	< 0.001
PL-7	10 (11.24)	2 (1.98)	0.020
PL-12	9 (10.11)	1 (0.99)	0.007
EJ	10 (11.24)	0 (0.00)	< 0.001
OJ	2 (2.25)	1 (0.99)	0.601
MAAs, n (%)			
Ku	4 (4.49)	9 (8.91)	0.360
PM-Scl-75	0 (0.00)	2 (1.98)	0.499
PM-Scl-100	0 (0.00)	1 (0.99)	1.000
Ro-52	57 (64.04)	19 (18.81)	< 0.001
Muscle weakness	56 (62.92)	80 (79.21)	0.020
Dermatologic manifestations			
Skin rash	30 (33.71)	40 (39.60)	0.490
Heliotrope rash	12 (13.48)	14 (13.86)	1.000
Gottron’s papules/signs	23 (25.84)	26 (25.74)	1.000
Calcinosis cutis	3 (3.37)	1 (0.99)	0.342
Mechanic’s hands	20 (22.47)	3 (2.97)	< 0.001

ASS, anti-synthetase syndrome; CTD, connective tissue disease; DM, dermatomyositis; IBM, inclusion body myositis; ILD, interstitial lung disease; IMNM, immune-mediated necrotizing myopathy; IQR, interquartile range; MAA, myositis-associated antibody; MSAs, myositis-specific antibody; PM, polymyositis; SD, standard deviation.

Most patients with ILD had ASS, while patients without ILD were more likely to have DM, overlap myositis, or IMNM. Concurrent autoimmune diseases or malignancy did not differ significantly between the two groups. However, mortality was significantly higher in patients with ILD (12 patients, 13.48%) compared to those without ILD (3 patients, 2.97%) with a *p*-value of 0.016.

The prevalence of various MSAs and MAAs differed between the two groups. Patients with ILD were more likely to have Ro-52 antibodies (64.04%) and most antisynthetase antibodies (e.g. Jo-1, PL-7, PL-12, and EJ antibodies), while patients without ILD were more likely to have TIF1γ and SRP antibodies (10.89%, 9.90%, respectively). There were no significant differences in the prevalence of other antibodies between the two groups.

In terms of clinical manifestations, muscle weakness was more common in patients without ILD (79.21%) compared to those with ILD (62.92%) with a *p*-value of 0.020. There was no significant difference in the prevalence of dermatologic manifestations between the two groups, except for mechanic’s hands, which were more common in patients with ILD (22.47%) compared to those without ILD (2.97%) with a *p*-value of < 0.001.

### Risk of ILD in different subgroups

In comparison to the reference group of PM, patients with ASS displayed a significantly elevated crude odds ratio (OR) of 67.79 (95% CI: 10.07-456.54) for the occurrence of ILD, which remained markedly increased after adjusting for potential confounding factors (age, gender, and concurrent autoimmune diseases), resulting in an adjusted OR of 52.28 (95% CI: 7.67-356.47). In contrast, other IIM subtypes such as DM, IMNM, and overlap myositis did not demonstrate a notable increase in ILD risk when compared to the reference group (Table 2).

**Table 2:** Risk of ILD in different IIM subgroups and myositis antibody categories

	Crude OR (95% CI)	*p*-value	Adjusted OR (95% CI)^**^	*p*-value
IIM subgroup (n)				
DM (52)	5.24 (0.84–32.81)	0.077^*^	4.78 (0.77–29.90)	0.095^*^
ASS (63)	67.79 (10.07-456.54)	< 0.001^*^	52.28 (7.67-356.47)	< 0.001^*^
IMNM (16)	0.87 (0.08–10.15)	0.912^*^	0.88 (0.08–10.10)	0.917^*^
Overlap myositis (44)	3.46 (0.54–22.38)	0.192^*^	2.80 (0.30-25.87)	0.365^*^
PM (14)	ref.	-	ref.	-
Antibody positivity (n) vs. negativity (n)				
Any MSAs (102 vs. 87)	7.01 (3.66–13.41)	< 0.001	6.84 (3.39–13.77)	< 0.001
Any MSAs and Ro-52 antibodies (53 vs. 136)	22.24 (8.26–59.93)	< 0.001	20.86 (7.63–57.08)	< 0.001
Anti-ARS antibodies (63 vs. 126)	15.60 (6.97–34.93)	< 0.001	14.64 (6.43–33.33)	< 0.001
Anti-ARS and Ro-52 antibodies (42 vs. 147)	40.00 (9.28–172.40)	< 0.001	38.39 (8.83–167.00)	< 0.001
Anti-MDA5 antibodies (12 vs. 177)	2.37 (0.69–8.16)	0.171	2.86 (0.75–10.86)	0.124
Anti-MDA5 and Ro-52 antibodies (5 vs. 184)	13.09 (0.54-316.51)	0.114^*^	10.09 (0.39-260.64)	0.163^*^
Anti-Ro-52 antibodies (76 vs. 113)	7.59 (3.92–14.71)	< 0.001	8.42 (4.14–17.13)	< 0.001

^*^Firth’s bias-reduced penalized-likelihood logistic regression

^**^Adjusted for age, gender, and concurrent autoimmune diseases

ARS, aminoacyl-tRNA synthetase; ASS, anti-synthetase syndrome; CI, confidence interval; DM, dermatomyositis; IMNM, immune-mediated necrotizing myopathy; MSAs, myositis-specific antibodies; OR, odds ratio; PM, polymyositis

When examining specific autoantibodies, the presence of any MSA was associated with a six-fold increased risk of ILD compared to those without any MSA (adjusted OR = 6.84, 95% CI: 3.39–13.77, *p* < 0.001). The risk further escalated to 20-fold for those who were dual-positive for MSA and anti-Ro-52 antibodies (adjusted OR = 20.86, 95% CI: 7.63–57.08; *p* < 0.001). For anti-ARS antibodies, their presence was associated with a 14-fold higher risk of ILD (adjusted OR = 14.64, 95% CI: 6.43–33.33; *p* < 0.001). This risk ILD substantially increased to 38-fold for patients who had both anti-ARS and anti-Ro-52 antibodies, compared to individuals lacking either of these antibodies (adjusted OR = 38.39, 95% CI: 8.83–167.00; *p* < 0.001). Regarding anti-MDA5 antibodies, patients had a slightly elevated but not significant risk of ILD (adjusted OR = 2.86, 95% CI: 0.75–10.86; *p* = 0.124) compared to those without anti-MDA5 antibodies. Similarly, dual positivity for anti-MDA5 and anti-Ro-52 antibodies also showed a non-significant risk (adjusted OR = 10.09, 95% CI: 0.39-260.64; *p* = 0.163). Finally, the presence of anti-Ro-52 antibodies alone was associated with more than an eight-fold increased risk of ILD compared to those without anti-Ro-52 antibodies (adjusted OR = 8.42, 95% CI: 4.14–17.13; *p* < 0.001) (Table 2). If IIM-mimics were included and the entire cohort was analyzed, similar results were observed. However, patients with anti-MDA5 antibodies had a slightly elevated risk of ILD (adjusted OR = 3.70, 95% CI: 1.27–10.78; *p* = 0.017) compared to those without anti-MDA5 antibodies (see Supplementary Table 2 for details).

### Sensitivity analysis of MSAs and ARS antibodies

Table 3 presents MSAs’ sensitivity in distinguishing IIMs from IIM-mimics using two models (Model 1 and Model 2). In Model 1, sensitivity was 0.54, correctly identifying 102 of 190 IIM cases, with a specificity of 0.94 for 25 of 414 IIM-mimic cases, resulting in an area under the curve (AUC) of 0.74. Model 2 achieved higher sensitivity at 0.58, correctly identifying 110 IIM cases. However, it had reduced specificity at 0.83 for 69 IIM-mimic cases, resulting in an AUC of 0.71. Overall, Model 2 displayed improved sensitivity, but lower specificity and AUC compared to Model 1 in distinguishing IIM from IIM-mimics. A similar sensitivity analysis for anti-ARS antibodies was shown in Table 4. In Model 1, sensitivity reached 0.33, accurately identifying 63 of 190 IIM cases, with exceptional specificity of 0.99 for 6 of 414 IIM-mimic cases, resulting in an AUC of 0.66. Model 2 exhibited slightly improved sensitivity at 0.34, correctly identifying 64 IIM cases, alongside notable specificity of 0.94 for 25 IIM-mimic cases, resulting in an AUC of 0.64. In summary, Model 2, while slightly more sensitive, displayed a trade-off in specificity and AUC compared to Model 1 when distinguishing IIM from IIM-mimics using anti-ARS antibodies.

**Table 3:** Sensitivity analysis of MSAs in model 1 and 2

	Model 1^*^	Model 2^**^
IIM	102 (53.68)	110 (57.89)
IIM-mimics	25 (6.04)	69 (16.67)
Sensitivity	0.54	0.58
Specificity	0.94	0.83
AUC	0.74	0.71

^*^Model 1: Autoantibodies were considered positive for moderate positive titers (++) or more.

^**^Model 2: Autoantibodies were considered positive for weak positive titers (+) or more.

MSAs, myositis-specific antibodies; AUC, area under the ROC curve.

**Table 4:** Sensitivity analysis of anti-ARS antibodies in model 1 and 2

	Model 1^*^	Model 2^*^
IIM	63 (33.16)	64 (33.68)
IIM-mimics	6 (1.45)	25 (6.04)
Sensitivity	0.33	0.34
Specificity	0.99	0.94
AUC	0.66	0.64

^*^Model 1: Autoantibodies were considered positive for moderate positive titers (++) or more.

^**^Model 2: Autoantibodies were considered positive for weak positive titers (+) or more.

ARS, aminoacyl-tRNA synthetase; AUC, area under the ROC curve.

### Clinical profiles in IIM patients with different MSAs and MAAs

Table 5 provides an overview of different MSAs and MAAs in our IIM patient cohort, accompanied by their corresponding clinical characteristics. The most frequently detected antibodies were Jo-1 (28 cases), followed by Ku (13 cases), TIF1γ, MDA5, PL-7 (12 cases each), and SRP (11 cases). The median age of onset was similar among MSA groups, ranging from 53 to 60 years, except for the SRP group, which had the youngest median age of onset at 39 years. The Jo-1 group had the highest female-to-male ratio among all MSAs.

**Table 5:** Clinical characteristics of all IIM patients according to antibody specificities

	Mi-2α	Mi-2β	TIF1γ	MDA5	NXP2	SAE1	Jo-1	PL-7	PL-12	EJ	OJ	SRP	Ku	PM-Scl 100	PM-Scl 75
Number	4	4	12	12	5	2	28	12	10	10	3	11	13	1	2
Age of onset (median)	60.0	55.0	60.0	60.0	53.0	66.5	55.0	67.0	55.0	54.5	73.0	39.0	46.0	46.0	58.5
Gender F: M	3:1	2:2	7:5	8:4	3:2	2:0	22:6	9:3	5:5	6:4	1:2	6:5	11:2	1:0	1:1
CK (U/L) (median)	8192.0	323.5	170.0	117.0	765.0	83.0	801.0	861.0	58.0	299.0	164.0	8535.0	1587.0	443.0	6259.5
LD (U/L) (median)	892.0	192.5	332.0	403.0	513.0	224.5	374.5	478.0	232.5	289.0	258.0	974.5	366.5	NA	1008.0
AST (U/L) (median)	384.0	30.5	54.0	74.0	41.0	28.0	60.0	105.5	24.5	114.0	25.5	244.0	67.0	NA	510.0
Malignancy	2	2	3	0	0	0	1	1	2	1	1	1	2	0	0
Mortality	0	1	2	5	0	0	0	1	1	4	0	0	0	0	0
Ro-52, n	1	0	3	5	2	0	22	5	6	9	0	4	3	1	0
Ro-52 (%)	25	0	25	41.7	40	0	78.6	41.7	60	90	0	36.4	23.1	100	0
ILD	0	1	1	8	4	1	23	10	9	10	2	1	4	0	0
Muscle weakness	4	3	8	8	3	0	20	7	6	6	0	11	10	1	1
Skin rash	3	1	12	11	3	1	7	3	2	4	0	2	2	0	0
Heliotrope rash	1	1	6	2	1	0	1	1	1	2	0	0	0	0	0
Gottron’s papules/signs	1	1	9	10	2	1	5	2	3	1	0	1	1	0	0
Calcinosis cutis	0	0	0	1	1	0	1	0	0	0	0	0	0	0	0
Mechanic’s hands	0	0	1	2	0	0	7	4	4	0	1	0	0	0	0

Both the Mi-2α and Mi-2β groups had two cases of malignancy out of four cases each, while the TIF1γ group had three cases of malignancy out of 12. The MDA5 group had the highest mortality rate, with 5 out of 12 cases succumbing to the disease.

The Mi-2α and SRP groups exhibited the highest CK levels (median: 8192 and 8535 U/L, respectively), LD levels (median: 892 and 974.5 U/L, respectively), and AST levels (median: 384 and 244 U/L, respectively). These findings were consistent with the fact that the Mi-2α and SRP groups had the highest proportion of individuals with muscle weakness, as 100% of cases in both groups tested positive for muscle weakness.

The TIF1γ group showed the strongest association with skin rash, with all 12 cases (100%) in this group presenting with this symptom. Additionally, substantial numbers of cases with skin rash were found in the MDA5 group (11 out of 12 cases) and the Mi-2α group (3 out of 4 cases). Gottron’s papules/signs were most common in the MDA5 group (10 out of 12 cases), followed by the TIF1γ group (9 out of 12 cases). Mechanic’s hands were most commonly associated with the PL-12 group (4 out of 10 cases), followed by PL-7 (4 out of 12 cases) and the Jo-1 group (7 out of 28 cases).

The Jo-1 group had the highest number of cases positive for Ro-52 antibodies (22 out of 28), but its proportion was lower than that of the EJ group (9 out of 10 cases). Additionally, the Jo-1 group had the highest number of ILD cases (23 out of 28), but its proportion was lower than that of the EJ group (10 out of 10 cases).

## Discussion

In this study, we investigated the association between various myositis autoantibodies and ILD in patients with IIM. The findings revealed that patients with IIM have a higher incidence of ILD, concurrent autoimmune diseases, and elevated levels of multiple autoantibodies. Notably, patients with IIM and ILD had a higher age of IIM onset and mortality rate than those without ILD. Certain autoantibodies, such as MSAs as a whole, anti-ARS, and anti-Ro-52 antibodies, were identified as being associated with an increased risk of ILD. Dual positivity for anti-ARS and anti-Ro-52 antibodies was found to confer the highest risk of ILD.

Approximately 20–40% of individuals diagnosed with IIM tested positive for the anti-Ro-52 antibody [28–32]. The Jo-1 antibody, which was the first anti-ARS antibody to be identified [6, 33], was also the initial one associated with a higher prevalence of anti-Ro-52 antibodies, exceeding 50%, in contrast to the overall rate of anti-Ro-52 positivity in IIM cases [28, 32]. The coexistence of autoantibodies against Jo-1 and Ro-52 might indicate a coupling effect in the development of autoimmunity [28, 34]. Other anti-ARS antibodies, such as PL-7, PL-12, and EJ, were also observed to have a comparably high frequency of anti-Ro-52 antibody presence, similar to Jo-1 [35, 36]. In our cohort, the prevalence of anti-Ro-52 antibodies among IIM patients with anti-ARS antibodies did not differ significantly from that observation.

In a previous study conducted during the era when ASS was classified within either DM or PM, researchers observed that anti-Ro-52 antibodies were linked to a 2.8-fold increased risk of pulmonary complications in dermatomyositis, while anti-Jo-1 antibodies were associated with a 3.9-fold higher risk of pulmonary disorders in polymyositis [20]. A recent study by Xing et al. focused on dermatomyositis, which certainly included some cases of ASS, identified anti-Ro-52 antibodies as a significant risk factor for the development of ILD with an odds ratio of 3.106. [21]. Another study by Vojinovic et al. that encompassed all five subtypes of IIMs demonstrated a significant association between anti-Ro-52 antibodies and ILD with an odds ratio of 3.97 [22]. The results of these studies were in line with our findings, which indicated that in patients with IIM, the presence of isolated anti-Ro-52 antibody positivity was associated with a more than eight-fold increased risk of developing ILD.

Regarding anti-Ro-52 antibodies in ASS, several studies have provided insights into their impact. Marie et al. conducted a study on IIM patients classified as anti-Jo-1 antibody positive ASS, revealing that the presence of anti-Ro-52 antibodies did not lead to an increased risk of ILD. However, within the subgroup of patients with ILD, those with anti-Ro-52 antibodies were more likely to experience symptomatic form of ILD [37]. Bauhammer et al. investigated IIM individuals with Jo-1 antibodies, indicative of Jo-1 antibody-associated ASS, and found that anti-Ro-52 antibodies were present in 43% of cases. Notably, the presence of anti-Ro-52 antibodies was significantly associated with the development of acute-onset ILD. Furthermore, those with high anti-Ro-52 antibody concentrations faced the highest risk [38]. Bozzalla-Cassione et al. examined the impact of anti-Ro-52 antibodies in patients with ASS involving five major anti-ARS antibodies (Jo-1, PL-7, PL-12, EJ, OJ) [39]. ASS was defined as the presence of at least one anti-ARS antibody along with one classic triad manifestation (arthritis, myositis, or ILD). The study found a significantly higher prevalence of ILD in patients who tested positive for anti-Ro-52 antibodies at the last follow-up. Shi et al. also conducted a study focusing on ASS involving these five major anti-ARS antibodies [40]. Although they did not find significant associations with ILD in general, their results showed that ASS patients with anti-Ro-52 antibodies had a higher incidence of rapidly progressive ILD (RP-ILD) than those without anti-Ro-52. Additionally, there was a statistically significant increase in RP-ILD prevalence among ASS patients with anti-PL7 antibodies compared to those without anti-PL7. Importantly, patients who tested positive for both anti-PL7 and anti-Ro-52 antibodies had a higher likelihood of experiencing RP-ILD compared to those without anti-Ro-52. While our study did not specifically assess RP-ILD, we did find an association between anti-ARS antibodies and an increased risk of ILD, consistent with established knowledge. Moreover, the presence of coexisting anti-Ro-52 antibodies along with anti-ARS antibodies was associated with an even higher risk, aligning with the findings of previous studies. These results are consistent with prior research suggesting that anti-Ro-52 antibodies are linked to ILD, including RP-ILD, in individuals with ASS.

Anti-MDA5 antibody positive DM has been associated with an unfavorable outcome, primarily due to the high prevalence of RP-ILD. Significant associations between anti-MDA5 antibodies and RP-ILD have been reported in Asian studies, while studies in Caucasian populations have shown variations in these associations [41]. Earlier studies investigating the clinical manifestations of anti-MDA5 positive DM have demonstrated a higher risk of ILD in patients with positive anti-MDA5 antibody compared to those with negative antibodies or other myositis antibodies [42–51]. This association remains true regardless of whether anti-MDA5 is linked to RP-ILD or not.

A recent study by Xing et al. on risk factors for ILD in DM patients found that anti-MDA5 antibodies were a significant risk factor for ILD development, with an odds ratio of 10.445 [21]. Although the study did not specifically address RP-ILD, it did find that individuals who tested positive for both anti-Ro-52 and anti-MDA5 antibodies experienced a poorer outcome compared to those who were positive for either anti-Ro-52 or anti-MDA5 antibodies alone. In a study by Wen et al. among IIM patients, anti-MDA5 antibodies were the most common MSAs, found in 25.4% of cases, and were significantly linked to RP-ILD. Patients with both anti-MDA5 and anti-Ro-52 antibodies also had the worst prognosis [52]. Our study, while not focusing on RP-ILD or outcomes among anti-MDA5 positive patients, did not find an elevated risk of ILD in individuals with anti-MDA5 antibodies compared to those without.

In a study by Xu et al. involving clinically amyopathic DM-associated ILD patients with positive anti-MDA5 antibodies, the presence of anti-Ro-52 antibodies increased the risk of RP-ILD, and those with both anti-MDA5 and anti-Ro-52 antibodies had a significantly lower survival rate than those with mild anti-MDA5 positivity alone [53]. In Gui et al.’s study on patients with IIM-associated ILD, anti-Ro-52 antibodies were linked to the development of RP-ILD and lower survival rates. This association remained consistent within the subgroup of patients with anti-MDA5 antibodies as well [54]. In Lv et al.’s study involving anti-MDA5 + DM patients, individuals with anti-Ro-52 positivity experienced a higher incidence of RP-ILD and increased mortality [55]. In You et al.’s study of anti-MDA5 + DM patients, they discovered that anti-Ro-52 antibody positivity as well as moderate to high anti-MDA5 antibody titers, among others, were independent risk factors for RP-ILD, and RP-ILD itself was identified as an independent risk factor for mortality [56]. In a cluster analysis study by Xu et al. on anti-MDA5 + DM patients, the cluster with a high RP-ILD risk was more likely to include patients with both anti-Ro-52 antibodies and high anti-MDA5 antibody titers and had the highest mortality rates [57]. However, our study did not find an increased ILD risk with dual positivity for anti-MDA5 and anti-Ro-52 antibodies. One possible explanation for this inconsistency is the relatively smaller sample size of MDA5-positive patients in our study compared to previous research, potentially limiting our ability to detect significant differences. Another hypothesis is that anti-Ro-52 antibodies may specifically elevate the risk of RP-ILD rather than ILD in general among anti-MDA5 positive patients. Further research with larger sample sizes and a focus on both ILD and RP-ILD may provide more insights into these associations.

Two criteria sets have been proposed for the diagnosis of ASS. Connors’ criteria only require the presence of one of the six ASS manifestations [25], while Solomon’s criteria demand either both major criteria, namely myositis and ILD, or one major criterion along with two of the three minor criteria, which include arthritis, Raynaud’s phenomenon, and mechanic’s hands (fever is not included) [58]. However, myositis may not necessarily occur in the initial stages of ASS. In a long-term observational study, the development of ILD before the onset of myositis was more frequently observed in patients with anti-PL-7/PL-12 antibodies than in those with anti-Jo-1 antibodies [59]. Longitudinal cohort studies have revealed that individuals with non-Jo-1 autoantibodies tend to exhibit a lower prevalence and less severe muscle involvement [60], and only 40% of non-Jo-1 patients receive a diagnosis of IIM at first visit [61]. Additionally, clinicians other than rheumatologists who may encounter IIM patients, such as dermatologists, neurologists, or pulmonologists, might not be adept at recognizing all six manifestations of ASS. Hence, the stricter Solomon’s criteria may not capture all possible ASS cases. As a result, we applied Connors’ criteria to identify cases of ASS.

The exact mechanisms underlying the association of anti-Ro52 antibodies with ILD are not fully understood, yet insights from Decker et al. and Chan’s reviews provide some clues [14, 15]. Ro-52 protein mediates intracellular antibody immunity by acting as a cytosolic Fc receptor for immunoglobulins bound to viral antigens. This process directs incoming antibody-virus complexes towards proteasomal degradation, thereby neutralizing the viruses and impeding infection. Additionally, Ro-52 protein functions as an E3 ubiquitin ligase, which is crucial for the ubiquitination of cellular proteins, including various interferon regulatory factors. These factors play a vital role in mediating type 1 interferons, which are essential for antimicrobial defenses, especially antiviral responses. Ro-52 protein can be induced by type 1 interferons, and its E3 ubiquitin ligase activity may establish a negative feedback mechanism. This mechanism regulates excessive production of type 1 interferons and prolonged activation of the immune system during antiviral responses, thereby mitigating the risk of autoimmune disease development. Based on evidence that anti-Ro-52 antibodies may play a direct pathogenic role in congenital heart block in neonatal lupus and that these antibodies from patients with primary Sjögren’s syndrome inhibit the E3 ligase activity of the Ro-52 protein, it is postulated that these autoantibodies might exacerbate proinflammatory signals mediated by type 1 interferons. Since Ro-52 protein is expressed more abundantly in the lungs than in other parts of the body and ranks among the most antigenic proteins in humans, the inhibition of Ro-52 protein’s regulatory activity could result in an overactive immune response and inflammation in the lungs.

The study has several limitations. Firstly, it is imperative to recognize its retrospective, single-center design, which could introduce selection bias and limit representation of the broader IIM patient population, potentially affecting the ability to detect significant associations, especially given the relatively modest sample size in specific MSA subgroups. Addressing these limitations necessitates further investigations with larger, more diverse cohorts. A second critical limitation relates to missing clinical and serological data for some patients, which might impact the results. Third, the study did not distinguish between different ILD subtypes or explore RP-ILD. It is vital to acknowledge the diversity of ILD, necessitating more comprehensive research into subtypes and their associations with autoantibodies, including links to RP-ILD. Additionally, observed discrepancies in the frequency of CT scans among patients with positive and negative myositis antibodies within the IIM group could introduce bias in estimating ILD risk. While efforts were made to mitigate this potential bias by confirming that most patients underwent chest CT scans before or concurrently with the myositis antibody test, some degree of bias may still be present. Lastly, the associations between autoantibodies and ILD were primarily identified through regression analyses, revealing potential risk factors but not causation. To understand the precise mechanisms, additional mechanistic studies are essential.

## Conclusions

In conclusion, this study has provided valuable insights into the clinical and serological aspects of ILD in patients with IIMs. We have identified specific risk factors, particularly the presence of anti-Ro-52 antibodies and anti-synthetase antibodies, which significantly increase the likelihood of ILD development in this patient population. The high prevalence of ILD in IIMs and its impact on patient outcomes underscore the importance of early detection and monitoring. The utilization of these autoantibodies as potential biomarkers for ILD risk can aid in timely intervention and personalized patient care. This research contributes to our understanding of IIM-associated ILD and paves the way for improved management strategies, ultimately enhancing the quality of life for individuals battling these complex autoimmune conditions. Further investigations into the mechanistic links between autoantibodies and ILD are warranted, offering promise for more targeted treatments and refined patient care in the future.

### Electronic supplementary material

Below is the link to the electronic supplementary material.

Supplementary Material 1

## Data Availability

No datasets were generated or analysed during the current study.
