# Supplementary material for: Association of anti-Ro-52 antibodies with occurrence of interstitial lung disease in patients with idiopathic inflammatory myopathy
Source: Arthritis Res Ther. 2024 Aug 22;26:152. doi: 10.1186/s13075-024-03382-x (PMC11340136; doi:10.1186/s13075-024-03382-x)
Supplement: Supplementary file 1 — Supplementary Material 1 [file 13075_2024_3382_MOESM1_ESM.docx]

**Supplementary material**

| Supplementary Table 1 Clinical and serological features of patients with IIM and IIM-mimics | | | |
| --- | --- | --- | --- |
|  | IIM (N = 190) | IIM-mimics (N = 414) | p-value |
| Age of onset (years), mean (SD) | 52.6 (15.4) | 54.1 (17.1) | 0.310 |
| Male, n (%) | 59 (31.05) | 135 (32.61) | 0.775 |
| CK (U/L), median (IQR) | 490.0 (136.0, 2862.0) | 81.0 (52.0, 147.0) | 0.363 |
| LD (U/L), median (IQR) | 365.0 (250.0, 599.0) | 198.0 (165.0, 259.0) | <0.001 |
| AST (U/L), median (IQR) | 67.0 (37.0, 176.0) | 27.0 (21.0, 37.0) | <0.001 |
| IIM subgroup, n (%) |  |  | - |
| DM | 52 (27.37) | - |  |
| ASS | 63 (33.16) | - |  |
| IMNM | 16 (8.42) | - |  |
| PM | 14 (7.37) | - |  |
| Overlap myositis | 44 (23.16) | - |  |
| IBM | 1 (0.53) | - |  |
| Concurrent autoimmune diseases, n (%) |  |  | <0.001 |
| None | 123 (64.74) | 184 (44.44) |  |
| Systemic lupus erythematosus | 15 (7.89) | 51 (12.32) |  |
| Primary Sjögren’s syndrome | 23 (12.11) | 65 (15.70) |  |
| Systemic sclerosis | 7 (3.68) | 16 (3.86) |  |
| Rheumatoid arthritis | 10 (5.26) | 19 (4.59) |  |
| Mixed CTD | 2 (1.05) | 5 (1.21) |  |
| Undifferentiated CTD | 10 (5.26) | 49 (11.84) |  |
| ANCA-associated vasculitis | 0 (0.00) | 3 (0.72) |  |
| IPAF | 0 (0.00) | 11 (2.66) |  |
| Other | 0 (0.00) | 11 (2.66) |  |
| ILD, n (%) | 89 (46.84) | 88 (21.26) | <0.001 |
| Malignancy, n (%) | 18 (9.47) | 50 (12.08) | 0.423 |
| Mortality, n (%) | 15 (7.89) | 19 (4.59) | 0.148 |
| MSAs, n (%) |  |  |  |
| Mi-2α | 4 (2.11) | 5 (1.21) | 0.473 |
| Mi-2β | 4 (2.11) | 4 (0.97) | 0.268 |
| TIF1γ | 12 (6.32) | 1 (0.24) | <0.001 |
| MDA5 | 12 (6.32) | 5 (1.21) | 0.001 |
| SAE1 | 2 (1.05) | 1 (0.24) | 0.234 |
| NXP2 | 5 (2.63) | 3 (0.72) | 0.128 |
| SRP | 11 (5.79) | 4 (0.97) | 0.001 |
| Jo-1 | 28 (14.74) | 1 (0.24) | <0.001 |
| PL-7 | 12 (6.32) | 2 (0.48) | <0.001 |
| PL-12 | 10 (5.26) | 1 (0.24) | <0.001 |
| EJ | 10 (5.26) | 0 (0.00) | <0.001 |
| OJ | 3 (1.58) | 2 (0.48) | 0.182 |
| MAAs, n (%) |  |  |  |
| Ku | 13 (6.84) | 19 (4.59) | 0.341 |
| PM-Scl-75 | 2 (1.05) | 6 (1.45) | 1.000 |
| PM-Scl-100 | 1 (0.53) | 0 (0.00) | 0.315 |
| Ro-52 | 76 (40.00) | 93 (22.46) | <0.001 |
| No MSA or MAA, n (%) | 61 (32.11) | 360 (86.96) | <0.001 |
| ASS, anti-synthetase syndrome; CTD, connective tissue disease; DM, dermatomyositis; IBM, inclusion body myositis; ILD, interstitial lung disease; IMNM, immune-mediated necrotizing myopathy; IPAF, interstitial pneumonia with autoimmune features; IQR, interquartile range; MAA, myositis-associated antibody; MSAs, myositis-specific antibody; PM, polymyositis; SD, standard deviation. | | | |

| Supplementary Table 2 Risk of ILD in different subgroups and myositis antibody categories | | | | |
| --- | --- | --- | --- | --- |
|  | Crude OR (95% CI) | p-value | Adjusted OR (95% CI)^**^ | p-value |
| IIM subgroup with IIM-mimics (n) |  |  |  |  |
| IIM-mimics (414) | 2.44 (0.42-14.20) | 0.321^*^ | 1.83 (0.29-11.38) | 0.517^*^ |
| DM (52) | 5.24 (0.84-32.81) | 0.077^*^ | 5.37 (0.81-35.53) | 0.081^*^ |
| ASS (63) | 67.80 (10.07-456.62) | <0.001^*^ | 73.94 (10.29-531.06) | <0.001^*^ |
| IMNM (16) | 0.87 (0.08-10.16) | 0.912^*^ | 0.79 (0.06-10.23) | 0.858^*^ |
| Overlap myositis (44) | 3.46 (0.54-22.38) | 0.192^*^ | 4.46 (0.62-32.22) | 0.138^*^ |
| PM (14) | ref. | - | ref. | - |
|  |  |  |  |  |
| Antibody positivity (n) vs. negativity (n) |  |  |  |  |
| Any MSAs (127 vs. 477) | 5.30 (3.50-8.04) | <0.001 | 6.09 (3.85-9.63) | <0.001 |
| Any MSAs and Ro-52 antibodies (57 vs. 547) | 17.28 (8.26-36.18) | <0.001 | 23.04 (10.48-50.63) | <0.001 |
| Anti-ARS antibodies (69 vs. 535) | 16.43 (8.53-31.62) | <0.001 | 20.93 (10.23-42.83) | <0.001 |
| Anti-ARS and Ro-52 antibodies (43 vs. 561) | 41.27 (12.57-135.50) | <0.001 | 55.26 (16.24-188.10) | <0.001 |
| Anti-MDA5 antibodies (17 vs. 587) | 2.81 (1.07-7.39) | 0.037 | 3.70 (1.27-10.78) | 0.017 |
| Anti-MDA5 and Ro-52 antibodies (5 vs. 599) | 27.28 (1.14-654.23) | 0.041^*^ | 19.25 (0.74-504.00) | 0.076^*^ |
| Anti-Ro-52 antibodies (169 vs. 435) | 3.63 (2.49-5.31) | <0.001 | 5.66 (3.59-8.92) | <0.001 |

*Firth’s bias-reduced penalized-likelihood logistic regression.

**Adjusted for age, gender, and concurrent autoimmune diseases.

ARS, aminoacyl-tRNA synthetase; ASS, anti-synthetase syndrome; CI, confidence interval; DM, dermatomyositis; IMNM, immune-mediated necrotizing myopathy; MSAs, myositis-specific antibodies; OR, odds ratio; PM, polymyositis
